# Mushrooms and Truffles: Historical Biofactories for Complementary Medicine in Africa and in the Middle East

**DOI:** 10.1155/2013/620451

**Published:** 2013-11-20

**Authors:** Hesham El Enshasy, Elsayed A. Elsayed, Ramlan Aziz, Mohamad A. Wadaan

**Affiliations:** ^1^Institute of Bioproduct Development (IBD), Universiti Teknologi Malaysia (UTM), Skudai, 81130, Johor Bahru, Johor, Malaysia; ^2^City of Scientific Research and Technology Application, New Burg Al Arab, Alexandrai 21934, Egypt; ^3^Bioproducts Research Chair, Department of Zoology, Faculty of Science, King Saud University, Riyadh 11451, Saudi Arabia; ^4^Department of Natural and Microbial Products, National Research Centre, Dokki, Cairo 12311, Egypt

## Abstract

The ethnopharmaceutical approach is important for the discovery and development of natural product research and requires a deep understanding not only of biometabolites discovery and profiling but also of cultural and social science. For millennia, epigeous macrofungi (mushrooms) and hypogeous macrofungi (truffles) were considered as precious food in many cultures based on their high nutritional value and characterized pleasant aroma. In African and Middle Eastern cultures, macrofungi have long history as high nutritional food and were widely applied in folk medicine. The purpose of this review is to summarize the available information related to the nutritional and medicinal value of African and Middle Eastern macrofungi and to highlight their application in complementary folk medicine in this part of the world.

## 1. Introduction 

From early stages of civilization, desert macrofungi in forms of mushrooms and truffles have been used as food and medicine. Originally, these types of organisms were associated with Mediterranean region and were first recorded as poem in Egyptian temples as follows: “Without leaves, without buds, without flowers: yet they from fruit; as a food, as a tonic, as a medicine: the entire creation is precious.” Thus, macrofungi were considered as food and medicine for royalty, and that no normal citizens were allowed to consume this precious food. During Greek and Roman eras, they were imported from Libya and sold in southern part of the European continent [1]. In the southern part of African continent, the nomadic people of Kalahari Desert used truffles for millennia [2]. 

Mushrooms are visible to the naked eye as they grow above the earth, whereas truffles grow underground in depth between 5 and 10 cm. Truffles are usually collected by specialists who have special skills and experience to explore this type of flora. Sometimes, truffle collectors use some animals such as pigs and dogs to discover this type of underground fungus. This is based on their high sensitivity to the characteristic truffle volatile compounds. Traditionally, mushrooms and truffles are taken as type of precious food and consumed either raw or cooked. In addition, they have been also applied as main component of folk medicine. This was based on the fact that they are rich source for proteins, amino acids, fatty acids, fiber, minerals, vitamins, terpenoids, sterols, flavor compounds, and carbohydrates as reported by many authors [3–5]. 

In general, not all types of macrofungi are able to grow in the harsh environmental conditions of desert. The term “Desert Macrofungi: Mushrooms and Truffles” is related to the nature and distribution of those species which can grow under arid and semiarid conditions. Thus, the geographical distribution of desert truffles in Africa and Middle East is related to countries around the Mediterranean such as Morocco, Algeria, Tunisia, Libya, Egypt, and Israel in addition to the countries of the Arabian Peninsula such as Jordan, Syria, Saudi Arabia, Iraq, Bahrain, and Kuwait. However, some types of desert truffles were also found in South Africa and Botswana [6]. Generally, the growth of desert truffles requires an annual rainfall range between 50 and 380 mm. In North Africa, good yields of truffle are usually obtained if the rainfalls range between 70 to 120 mm. In addition, the time, quantity, and distribution of the rainfall play an important role in the quality of desert macrofungus growth. For example, to obtain good truffles in North African and Middle Eastern regions, it needs to get rainfall no later than the beginning of December whereas, in southern Europe it should not be later than the beginning of October [7]. 

For centuries, it was proposed that most of the wild macrofungi are not cultivable. However, with the increased knowledge of mushroom and truffles physiology, nowadays, it is possible to cultivate many types of macrofungi. Mushrooms were successfully cultivated in green houses and in submerged culture fermentation. Whereas, based on the symbiotic behavior of truffles as typical ectomycorrhiza, they were cultivated in soil with their host plant in truffle green houses in semiarid area [6, 7]. With the rapid growth of bioprocess technology industries, it was possible to cultivate many macrofungi in submerged culture under fully controlled conditions to produce the desired biotherapeutic compounds in high concentrations, in shorter production time, and under fully sterile conditions according to cGMP [8–10]. 

This review outlines the current status of knowledge on the macrofungi bioactive compounds and their applications in complementary and alternative medicine in different African and Middle Eastern cultures. 

## 2. Type and Identification

Different types of desert epigeous macrofungi (mushrooms) and hypogeous macrofungi (truffles) are considered as natural flora in the Middle East and the African desert. Mushrooms are characterized by their distinguished structure of stalk and their fragile and soft feature. Truffles have no stalk and no gills with firm, dense, and woody feature. The name truffles/*terfezia* comes from the word “Terfass,” which is an Arabic word describing hypogeous desert fungus. Truffles are also known locally in North Africa and Arabian Peninsula under other names such as “Al-Kamaa” or “Al-Fag'a.” Taxonomically, truffles are ascomycetes fungi belonging to the genus *Tuber*, the family Tuberaceae, and the order Pezizales [11] and are distributed in six pezizalean families: Glaziellaceae, Discinaceae, Morchellaceae, Helvellaceae, Tuberaceae, Pezizaceae, and Pyronemataceae [12]. In general, most of known desert truffles species belong to genera such as *Terfezia, Delastreopsis*,* Balstonia, Delastria, Leucangium, Mattirolomyces, Phaeangium Picoa, Tirmania*, and *Tuber* [6]. Ecologically, desert truffles are symbiotic microorganisms and they establish ectomycorrhizal symbiotic relationship with species of *Helianthemum *genus. Therefore, the distribution of desert truffles is limited not only by the environmental conditions but also by the availability of the host plant. For example, in Arabian Peninsula, only three types of truffles belonging to *Terfezia*, the two black-dark brown colored truffles *Terfezia claveryi *and *Terfezia boudieri* (known locally as Ikhlasi), and the white-cream colored truffle belonging to *Tirmania nivea* (known locally as Zubaidi) are available in this region [13]. In addition, another species of truffle *Phaeangium lefebvrei* is commonly known as bird truffle “Faga Al toyoor or Hopper” and is mainly consumed by birds. In North Africa, in addition to the previous local names, many other names are also given for truffles such as “Nabat Al Radh, asqal, Bidat El Ardh and Banat Ober” [14]. 

Mushrooms spread in wide area in Africa and Middle East. The most common types of mushrooms belong to *Agaricus *and *Pleurotus* sp. (Basidiomycetes). In North Africa, different types of *Pleurotus *sp. were found only for few days after rainy season. Based on its morphological structure and physiology, mushrooms are more sensitive to high temperature and dry conditions compared to truffles. The tropical and subtropical regions of Africa are characterized by higher mushroom diversity compared to North Africa.  Table 1 shows some examples of different types of mushrooms and truffles in Africa and Middle East. 

## 3. Nutritional Value

Desert macrofungi are rich source of different types of essential nutrients, and thus their nutritional value and chemical profile were studied and reviewed by many authors [15]. The chemical analysis of desert truffles showed that the dry matter is composed of up to 60% carbohydrates, 20–27% protein, 3–7.5% fat (unsaturated and saturated fatty acids), 7–13% fiber, and 2–5% ascorbic acid [12]. Another study for chemical profiling of three Iraqi truffles (*Terfezia claveryi, Tirmania nivea*, and* Tirmania pinoyi*) showed that the carbohydrate concentration in dry matter ranged between 16.6 and 24.8%, protein content ranged from 8.1 to 13.8%, phosphorus from 9.7–25.5%, and ash 4.9–5.9% [16]. Another research showed also that the chemical composition of truffle is highly strain specific and the white desert truffle *Tirmania nivea* (Zubaidi) was higher in protein, fat, and crude fiber content compared to other two types of desert truffles belonging to black truffle group (Gibeah and Kholeissi) [17]. However, the same study showed also that all essential amino acids were present in all three truffles including the sulphur amino acids (methionine, cysteine, tryptophan, and lysine) which are usually the limiting amino acids in many foods of plant origin. In addition, different studies demonstrated also that truffles are rich source of essential minerals such as Si, K, Na, Ca, Mg, Mn, Fe, Al, Cu, and Zn [15]. 

Unlike truffles, the dry content of mushrooms is usually in the range between 60 and 140 g/kg. In most types of mushrooms, carbohydrates and crude proteins are the main two components. The composition of mushroom fruit bodies is very rich with carbohydrates, and its concentration is ranging between 20 up to more than 70% of the dry weight and is highly dependent on mushroom strain. Glucose, mannitol, and trehalose, their derivatives and oligosaccharides, are the main polysaccharides and share very low percentage in fruit body dry weight. Unlike plants which store polysaccharide in the form of starch, mushrooms store polysaccharides in the form of glycogen and usually contribute to about 5–10% of dry matter [5]. Chitin, a water insoluble polysaccharide, contributes to up to 80–90% of mushroom cell wall in addition to other components such as hemicelluloses and pectin [18]. Mushrooms contain also high content of insoluble fiber which also increases its nutritional value. On the other hand, mushrooms are very rich in proteins and usually comprise about 30% of dry weight. Protein distribution is usually changeable during the fungal development. It was reported that, for the widely distributed oyster mushroom (*Pleurotus ostreatus*), the highest crude protein content of about 36% with highest digestibility (92%) is mainly found in fungal cap of diameter ranging between 5 and 8 cm [19]. In addition to the high fungal protein content, the proportion of essential amino acids makes it of higher nutritional value compared to plant proteins [5]. The mushroom lipid content ranges between 2 and 6% of dry weight. The polyunsaturated linoleic acid, the monounsaturated oleic acid, and the saturated palmitic acids are the main components of mushroom fatty acids. Other branched chain acids and hydroxyl fatty acids are also present in some mushrooms but in very low concentrations [20, 21]. Mushrooms also include ash (5–12% of dry matter) and are rich in essential elements such as sodium, potassium, calcium, magnesium, phosphorus, and sulfur. Moreover, mushrooms also include many essential trace elements such as manganese, cupper, and selenium. The presence of chromium and selenium in acceptable concentrations (few mg per kg dry matter) increases the nutritional value of mushrooms and is considered as potential source of organic selenium/chromium in diets and food supplements [5]. 

## 4. Volatile Organic Compounds

In general, macrofungi are characterized by their ability to produce wide range of volatile compounds which gives the characteristic flavor of this group of organisms. The characteristic aroma compounds of fruiting bodies are often used for strain nomenclature in both Latin and local names to indicate special aromatic characteristics. For example, attributes like *butyrace-* (butter like), *odor-losm-* (fragrant), *delicat-* (delicious), *olid-* (ambrosial, *suav-* (sweet), and *nidoros-* (pungent) point out certain tastes and aromas [22].

Truffles are typical ectomycorrhizal fungi and thus must grow in association with host plant. The hypogeous fruiting bodies of this type of organisms are characterized by their strong aroma which helps the truffle hunters to find them in the soil. This complex aroma comes from easily evaporated low molecular weight carbon compounds which have common name as volatile organic compounds (VOCs). These compounds play crucial ecological role in the recognition between the fungus and the host plant and also regulate the symbiosis process. In addition, these compounds play another role in the interaction between the truffles and nonhost plant as well. Nowadays, more than 200 VOCs and many nonvolatile compounds have been isolated and identified from truffles fruiting bodies. Truffles VOCs include fatty acids, terpenoids, aromatic compounds, and sulfur containing compounds [23]. On the other hand, mushroom volatile flavors and VOCs are one of the fungal characteristics which sometimes determine their commercial value. In general, the flavor and special aroma of certain mushrooms are usually a combination of different VOCs. The chemical profile of volatile compounds of *P. ostreatus* includes C_8_ compounds such as oct-1-ene-3-ol, octan-3-ol, octan-3-one, octanal, oct-1-en-3-one, and octan-1-ol in addition to benzaldehyde (almond odor), benzyl alcohol (sweet spicy odor), and monoterpenes like linalool and linalool oxide. These all together make the characteristic aroma and pleasant flavor of this mushroom [24]. In other mushrooms like *Termitomyces schimperi*, which is common fungus in many African countries, the VOCs of fresh fruiting bodies include more than twenty-four compounds. They include oct-1-en-3-ol, 2-phenylethanol, and hexanal. It was also interesting that these VOCs were almost the same in both of caps and stems of this fungus [25]. 

However, these characteristic fungal VOCs are important during fungal life cycle, not only to increase their market value but also to contribute to the nutritional and medicinal values of macrofungi.

## 5. Functional Chemical Compounds of Health Value

### 5.1. Antioxidants

Macrofungi are well known to contain different bioactive polyphenolic compounds. These compounds act as effective antioxidants based on their excellent ability to scavenge free radicals and act as reducing agents. Thus, it was proposed by many authors that there are strong correlations between the antioxidant activities of certain type of fungi and the type and concentration of polyphenolic compounds. Different groups of desert truffles and mushrooms showed strong antioxidant activities based on their high polyphenolic and ergosterol contents [13]. In the study of Villares et al. [26], different types of bioactive organic compounds showing antioxidant activities were isolated from *Tuber *sp. These include, ergosterol such as ergosteryl ester, wide range of phenolic acids such as gallic, homogentisic, protocatechuic, *p-*hydroxybenzoic, and *o-* and *p-*coumaric acids, and other phenolic derivatives such as 3,4-dihydroxybenzaldehyde. The study of Al-Laith [13] showed also that the famous white desert truffle *T. nivea* showed high antioxidant activities. It was claimed that the fungal antioxidant capacity is attributed to the presence of various chemicals such as ascorbic acid, carotenoids, esterified phenolics, and free- and nonflavonoid phenolics and flavonoids. Another research showed that the small truffle *Picoa lefebvrei* is also among the most attractive mushrooms in folk medicine based on its high antioxidant properties [27]. 

### 5.2. Antimicrobial Activity

Since the early 20th century, fungi are known for their highly potent antimicrobial secondary metabolites. However, the antibacterial and antiviral activities of desert truffles were first studied in 1980s by Al-Marzooky [28] who investigated the *in vitro* biological activities of all aqueous polar and nonpolar extracts of *T. claveryi*. This extract exhibited good wide spectral antimicrobial activities especially against the trachoma causing disease *Chlamydia trachomatis*, stomach ulcer, and open cut. Another research demonstrated that the use of aqueous extract of *Terfezia claveryi *inhibited the growth of pathogenic bacteria such as *Staphylococcus aureus *[29] and *Pseudomonas aeruginosa *[30]. The antimicrobial activity of *Terfezia* was due to the production of small molecular weight peptide antibiotics [29]. In addition to the wide spectrum antibacterial activities, *Terfezia* extract possessed also antiviral activities [16]. Another research done by Dib-Bellahouel and Fortas [31] showed also that the ethyl acetate extract of *Tirmania pinoyi* exhibited potent antimicrobial activities against the G+ve bacteria *B. subtilis *and *S. aureus.* These all together make mushrooms and truffle important candidates in complementary medicine [32].

### 5.3. Immunomodulators and Antitumors

Immunomodulators or Biological Response Modifiers (BRMs) are compounds capable of interacting with the immune system to upregulate or downregulate specific aspects of the host response. Some of these compounds exhibiting also anticancer activities were isolated from different types of macrofungi [8, 33]. Different types of BRM were applied as immunotherapeutic agents and showed significant activity as potent anticancer compounds in both *in vitro* and *in vivo* models [34–36]. Of the two main types of macrofungi, mushrooms are a common source of producing this type of compounds naturally. BRMs were isolated from different mushrooms parts such as fruiting bodies, stalk, spores, and mycelium in addition to fermentation broth when cultivated in submerged culture. In some research, BRMs were applied in combination with conventional chemo- and radiotherapy during cancer treatment to increase their efficiency. In general, according to their chemical structure, mushroom BRMs were classified into four main categories: lectins, terpenoids, polysaccharides and their peptides, and fungal immunomodulator proteins (Fips) [8]. Since the discovery of the potent anticancer polysaccharide lentinan (derived from *Lentinus edodes)* by Ikekawa and his group in late 1960s, mushrooms polysaccharides became one of the most interesting topics of research for the discovery and development of new anticancer drugs. [37]. Most of the known mushroom bioactive polysaccharides have various branching types of (1→3)- and (1→6)-*β*-D-glucan and polysaccharide protein complex [38]. However, the biological activity of mushroom polysaccharides is highly dependent on the length and branching of the chain, chain rigidity, and helical conformation [39]. Pleuran (from *Pleurotus ostreatus*), Lentinan (from *Lentinus edodes*), Grifolan (from *Grifola frondosa*), Krestin-PSK (from *Polystitcus versicolor*), and Scleroglucan (from* Sclerotina sclerotiorum*) are the most famous bioactive polysaccharides from mushroom origin [8, 40–44]. 

### 5.4. Other Medical and Cosmeceutical Applications

The medicinal value of African and Middle Eastern macrofungi is not limited to the above mentioned applications. It was also reported that both truffles and mushrooms have also potential applications as hepatoprotective, cholesterol and sugar lowering, and anti-inflammatory agent [15, 32, 38, 42]. For example, the extract of the known desert truffle (*T. claveryi*) showed powerful hepatotoxic activity against the known hepatotoxin carbon tetrachloride [45]. In addition to the known and the well-studied medical applications, different mushroom extracts were applied in cosmetic products such as moisturizers, skin antifade and antiaging agents, skin revitalizers, and whitening cream [46]. 

## 6. Therapeutic Values and Ethnomycological Applications

The total number of mushrooms on earth is expected to be 140,000, among which only 10% (about 14,000) is known [47, 48]. It is strongly believed that a large number of the unknown species to be discovered may be present in Africa. Generally, there is little or no information about the therapeutic and medicinal uses of these mushrooms in Africa. Primarily, this may be attributed to the fact that this information is scattered in a multitude of sources which are not easily accessible to the international English-speaking community [49]. Secondly, unlike Japan and China, where the knowledge on medicinal applications of edible mushrooms has been documented [50], most of the information on indigenous applications of African mushrooms had been passed orally from one generation to the other [51]. Nowadays, the available knowledge on the medicinal applications of African mushrooms comes from elder people selling those [52] as well as from some ethnobotanical monographs summarizing data for a particular region of the world [53–56].

The variation of climatic conditions within the continent is directly reflected on the prevalence of mushrooms in different geographical regions. Accordingly, medicinal applications may differ not only between different countries, but also between different ethnic groups inhabiting the same country. Mushrooms belonging to species of *Termitomyces, Pleurotus, Lentinus, Lenzites, Trametes, Ganoderma, Pycnoporus, Coriolopsis*, and *Calvatia* have been reported to be used in folk medicine in Nigeria [48, 57, 58]. On the other hand, mushrooms of the species *Termitomyces, Agaricus, Boletus, Pleurotus, Cantharellus, Macrolepiota, Ganoderma*, and *Geastrum* have been reported in Tanzania [59, 60]. Moreover, Kamatenesi-Mugisha and Oryem-Origa [61] have reported that the ethnomycological application of toadstool mushrooms from the family Tricholomataceae has been documented in western Uganda. In Burkina Faso, the application of the mushroom *Parkia biglobosa* has been also reported [62].

Mushrooms have been used in sub-Saharan Africa during the Paleolithic period (7000–9000 B.P.), where their application has been traditionally related to mysticism [57, 63]. Historically, the first reports record the application of mushroom as a hallucinogenic agent by the people of the Yoruba tribe in Nigeria [64]. Additionally, Oso [52] reported that the Yoruba traditional doctors applied a medicinal preparation of *Termitomyces microcarpus* for the treatment of gonorrhea. The preparation was administered orally by pounding a large quantity of the mushroom fruiting bodies with the pulp of the fruit *Cucurbita pepo *Linn., the leaves of *Cassia alata* Linn., and other ingredients. They also used oral administration of *Calvatia cyathiformis* in a ground form with other herbal ingredients to treat a disease known in Yoruba as *Masomaso*. This disease is believed to prevent pregnancy, in which the woman begins to discharge effluvium through her vagina. Additionally, they used *C. cyathiformis *ground with *Daldinia concentrica *as a remedy for leucorrhoea, where the patient woman washes her vagina at predetermined intervals.

Recently, Akpaja et al. [51] and Ayodele et al. [65] reported different ethnomycological applications of mushrooms used by the people of the Igbos in South East and the Igalas in North central Nigeria. They reported that the Igbo and Igala tribes have used *Pleurotus tuberrigium* to overcome headache, stomach pain, fever, cold and constipation; *Lentinus squarrosulus *for treating mumps and heart diseases; *T. microcarpus* for treating gonorrhea; *Calvatia cyathiformis* for treating leucorrhea and bareness; *Ganoderma lucidum* for treating arthritis and neoplasia; *G*. *resinaceum* to reduce blood sugar level (hypoglycemic) and to overcome liver diseases (hepatoprotector); *G. applanatum* used as an antioxidant and anti diabetes; *Volvariella volvaceae *as an antibiotic and antineoplastic; and *Daldinia concentrica *for treating stomach ulcers and skin diseases. 

Mdachi et al. [60] reported that in Tanzania and most African countries, some wild mushrooms have been used in traditional medicine. They also reported that in some rural areas of Tanzania, a mushroom soup is provided to mothers after child delivery to enhance fast recovery, while other mushroom species are used as medicines for stomach and heart diseases. In addition, *Ganoderma *species has been used to treat sick cows, while some puffball mushrooms are traditionally used for wound healing in the Kilimanjaro region of Tanzania.

In 2007, Kamatenesi-Mugisha and Oryem-Origa [61] investigated the effect of medicinal plants used by traditional medical practitioners in inducing labor, namely, uterine contractions, during childbirths in and around Queen Elizabeth Biosphere Reserve in Uganda. They found that native women use toadstool mushroom from the family Tricholomataceae to induce labor during childbirth by enhancing uterine contractions.

Moreover, Beiersmann et al. [62] reported that young mothers in Burkina Faso use mushroom to treat a respiratory distress syndrome, locally known as *Dusukun yelema*, of which 80% are due to malarial acidoses. They apply the ashes of burned mushroom (*Parkia biglobosa*) onto the child's chest.  Table 2 summarizes some of the ethnomycological applications of mushrooms reported in different African countries.

In a recent study, Tibuhwa [59] explored the dietary, therapeutic, and ethnomycological applications of wild mushrooms in communities living around Ngorongoro and Serengeti National Park in Tanzania. The study aimed at exploring the actual taxonomy knowledge of the Kurya and Masai tribes and at developing a baseline data which can contribute to establishing mushroom traditional uses depository. The study revealed that most of the Kurya tribe people use wild mushrooms as either foodstuff or as tonic. Among the mushrooms used for therapeutic applications, they used *Termitomyces titanicus, T. letestu, T. eurhizus*, and *T. auranticus* for the treatment of different intestinal problems, for example, pain, ulcer, constipation, and stomach ache. *Termitomyces microcarpus* was found to be used as immune boosting agent and is given to sick people to speed up their recovery as well as lactating mothers. The study also revealed that both tribes have different manners in using the same mushroom.  Table 3 summarizes some of the ethnomycological knowledge on mushroom utilization by both tribes.

Truffles are hypogeous fruiting bodies of the ascomycetous fungi living symbiotically with soil plant roots [66]. Truffle species have a wide range of host plant species, require a calcareous soil, and have different geographical distribution [67, 68]. They have been found through Europe [69], especially in Italy, France, and Spain and throughout China [70], Australia [71], North Africa and sub-Saharan countries [72, 73], and the Middle East [14, 74]. 

Truffles are widely appreciated as a costly delicacy as well as for their organoleptic properties, especially aroma [67]. Additionally, truffles have been used to promote health and to prevent and to treat several diseases. They have been reported to possess anti-inflammatory, immunosuppressing, and anti-carcinogenic properties [13, 75], antioxidant properties [26, 76], and antimicrobial activities [29, 77, 78].

Truffles have been reported in many African countries [79–81]. Desert truffles have been traditionally used by the native people of the Kalahari in southern Africa for millennia [2, 82], as well as by the Saharan natives [73, 83, 84]. Truffles belonging to the species of *Terfezia* and *Tirmania* have been generally eaten by the native people of north Africa and the Middle East from prehistoric times. The Khoisan people (sometimes called the Bushmen or San) have used truffles from the species of *Kalaharituber, Eremiomyces*, and *Mattirolomyces* [82].

Contrary to mushrooms, less attention has been paid to the indigenous information about medicinal truffles in Africa. The available information comes from some amateurs and foreign tourists' documentation, as well as from knowledge accumulation passed orally from generation to the next [14]. Omer et al. [73] reported that desert truffles (*Tirmania *and *Terfezia *spp.) have been utilized in folk medicine for the treatment of ophthalmic diseases and as aphrodisiac agents. Moreover, the same truffles have been also reported to be used in folk medicine in sub-Saharan Africa and Middle East to treat skin and eye diseases, for example, Trichoma [16, 28]. Traditionally, desert truffles have been used as folk medicine in the Arabian countries over two millennia without any known complications [85]. In these countries, boiled truffle water extract has been recommended by the Bedouins for the treatment of trachoma, one of the earliest recorded eye diseases by the World Health Organization.

Spiritually, the Khoisan hunters in the Kalahari desert believe that desert truffles (*kuuste or n'xaba*) counteract the effects of poisoned arrows in shot animals [82], and until the animal is confirmed to be dead, the hunter can take no food or drink other than water. This is due to the fact that they believe that if the archer eats food, the wounded animal will regain its health and escape. Moreover, the hunters keep a piece of *kuuste* to eat as an antidote in case of being accidently wounded by a poisoned arrow.

## 7. Conclusions and Future Prospects

Africa and Middle East are very rich regions of unique types of macrofungi. Both truffles and mushrooms of this area of the world produce wide variety of interesting bioactive compounds of high medical value and were used for millennium in the treatment of different diseases. The main drawbacks for their application in modern medicine and for production in industrial scales are based on four main facts. First, most of these types of organisms are not cultivable in green house and thus their availability is seasonal and highly affected by climate change. The second fact is the wide variability of the bioactive ingredient contents which are highly dependent on collection time, procedure, season, and environment. Third, based on the chemical composition of both of mushrooms and truffles, they have high capacity to accumulate high concentration of heavy metals and radioactive isotopes. Thus, special consideration should be taken into account when collected from polluted areas. The fourth fact is the lack of standard testing protocols to guarantee the quality and the efficacy of the fungal product. Thus, more research is required to solve the above mentioned problem to increase the use of wild macrofungus in medical applications. This will change in part the current medical practice using chemically synthesized compounds of many side effects. 

## Figures and Tables

**Table 1:** Some examples of different types of wild macrofungi in Africa and in the Middle East region.

	Countries	References
Type of truffle		
*Terfezia boudieri *Chatin	Libya	[72]
*Terfezia claveryi *	Saudi Arabia, Bahrain, Iraq, Egypt, and Jordan	[29, 45]
*Terfezia pfeilii *	South Africa	[86]
*Terfezia * sp.	Tunisia	[87]
*Tirmania nivea *	Saudi Arabia, Morocco, Bahrain, Egypt, and Kuwait	[13, 16, 73, 88]
*Phaeangium lefebvrei *	Bahrain, Saudi Arabia, Egypt, and Kuwait	[14]
*Choiromyces echinulatus *	South Africa	[86]
*(Eremiomyces echinulatus) *		[82]
*Picoa juniper *	Tunisia	[89]
*Picoa lefebvrei *	Tunisia	[89]
*Kalaharituber pfeilii *	South Africa, Botswana	[82]
Types of mushrooms		
*Pleurotus * sp.	Almost in all African and Middle Eastern countries	[51]
*Agaricus * sp.	Almost in all African and Middle Eastern countries	
*Lentinus * sp.	Cameroon, Nigeria	[90]
*Russula * sp.	Madagascar, Zimbabwe	[91, 92]
*Cantharellus * sp.		
*Afroboletus luteolus *		
*Termitomyces * sp.	Zambia, Zimbabwe, Mozambique, Tanzania, Zaire, and Benin	[93]
*Lactarius * sp.		[94]
*Cantharellus * sp.		[95]
*Amanita zambiana *		
*Schizophyllum commune *	Ethiopia	[96]
*Chlorophyllum * sp.	Burkina Faso	[97]
*Phlebopus * sp.		

**Table 2:** Ethnomycological applications of mushrooms in different African countries.

Mushroom	Country	Ethnomycological applications	Reference
*Pleurotus tuber-rigium*	Nigeria	Treatment of headache, cold, fever, stomach ache, and constipation	
*Lentinus squarrosulus *	Nigeria	Treatment of mumps and heart diseases	
*Termitomyces microcarpus*	Nigeria	Treatment of gonorrhea	
*Calvatia cyathiformis*	Nigeria	Treatment of leucorrhea, bareness, and hiccups	
*Ganoderma lucidum*	Nigeria	Treatment of arthritis and neoplasia	
*G. resinaceum *	Nigeria	Lowering blood sugar level and protecting liver cells	[48]
*G. applanatum*	Nigeria	Antioxidant and used for lowering blood sugar level, as well as antihypertensive	
*Schizophyllum commune *	Nigeria	Treatment of diabetes	
*Volvariella volvaceae *	Nigeria	Antibiotic and antineoplastic	
*Auricularia auricular *	Nigeria	Treatment of hemorrhoids and hemoptysis	
*Daldinia concentrica *	Nigeria	Treatment of stomach ulcer and upset, skin disease, and whooping cough and prevention of excessive growth of fetus to ease the delivery	
*Polyporus officinalis *	Nigeria	Treatment of hernia, cough, and catarrh	

Soup of different wild mushrooms	Tanzania	Promote quick recovery of mothers after childbirth	[56]
*Ganoderma *spp.	Tanzania	Treatment of sick cows	[60]
Puffball mushrooms	Tanzania	Wound healing	[60]
*Termitomyces microcarpus *	Tanzania	Health promoter and inducer of breast lactation	[59]
*Termitomyces titanicus *	Tanzania	Treatment of abdominal pain, stomach ache and ulcers, and constipation	[59]

Toadstool mushroom	Western Uganda	Induce labor during childbirth	[61]

*Parkia biglobosa *	Burkina Faso	Treat respiratory distress syndrome resulting from malarial acidoses	[62]

**Table 3:** Ethnomycological knowledge on mushrooms utilization by the Kurya and Maasai tribes around Ngorongoro and Serengeti National Park, Tanzania, modified from [59].

Species	Ethnic name		Traditional uses	
	Kurya	Masai	Kurya	Masai
*Termitomyces microcarpus *	Bitoghose	Not known	Food: improve healthy to long-ill people and lactating mothers	Not known
*T. titanicus *	Lyugu	Ormambuli	Food: tonic for various gastrointestinal problems	Few know it as tonic for various gastrointestinal problems
*T. aurantiacus *	Nyankobhiti	Ormambuli	Food: tonic for stomach aching	Not known
*T. clypeatus *	Vihungumururyo	Ormambuli	Food	Not known
*T. eurhizus *	Amanyegiswa	Ormambuli	Food	Not known
*T. le-testui *	Lyugu	Ormambuli	Food	Few know it as tonic for various gastrointestinal problems
*T. mammiformis *	Bitoghose	Ormambuli	Food	Not known
*T. umkowaan *	Amughu	Ormambuli	Food	Few know it as tonic for various gastrointestinal problems
*T. tylerianus *	Bitoghose	Ormambuli	Food	Not known
*T. striatus *	Bitoghose	Ormambuli	Food	Not known
*Agaricus campestris *	Bitoghose	Ormambuli	Food	Not known
*Macrolepiota procera *	Binyankorogoto	Not known	Healing wounds	Not known
*Ganoderma boninense *	Binyankorogoto	Not known	Treat wound and skin infections	Not known
*Geastrum saccatum *	Uiborinyiti	Not known	Subject bees into anaesthesia state	Subject bees into anaesthesia state
*G. triplex *	Uiborinyiti	Not known	Subject bees into anaesthesia state	Subject bees into anaesthesia state
